# Investigation of the Prevalence of Non‐ABO Blood Group Antigens in Infertile Women Referred to University Hospitals in Iran: A Cross‐Sectional Study

**DOI:** 10.1002/hsr2.73285

**Published:** 2026-10-08

**Authors:** Zeinab Eftekhar, Mohammad Ali Jalali Far, Mehdi Torabizadeh, Najmaldin Saki, Roshan Nikbakht

**Affiliations:** ^1^ Thalassemia & Hemoglobinopathy Research Center, Health Research Institute Ahvaz Jundishapur University of Medical Sciences Ahvaz Iran; ^2^ Golestan Hospital Clinical Research Development Unit Ahvaz Jundishapur University of Medical Sciences Ahvaz Iran; ^3^ Fertility and Infertility Research Center Ahvaz Jundishapur University of Medical Science Ahvaz Iran

**Keywords:** agglutination, blood group antigens, infertility, phenotype, serological tests

## Abstract

**Background and Aims:**

Infertility nowadays is considered a disease and a significant global health issue. Despite extensive research, the cause of approximately 15% of infertility cases remains unknown. Additionally, despite the high financial and emotional costs associated with infertility treatment, there is no single universal method that definitively leads to pregnancy and the birth of a healthy baby. This study investigates the prevalence of minor blood group antigens in infertile and fertile women in Iran, contributing to the understanding of blood group distributions in this population.

**Methods:**

Blood samples from 60 infertile women and 65 fertile women were analyzed for antigens in the Rh, MNS, P1PK, Kell, Duffy, Kidd, and Lewis blood group systems using manual serological agglutination tests and gel card methods. Finally, the results were analyzed using SPSS Statistics version 27.

**Results:**

The frequencies of the studied antigens were as follows: M (83.3%), N (55%), S (60%), Jka (75%), Jkb (66.7%), P1(76.7%), Fya (66.7%), Fyb (73.3%), Lea (16.7%), Leb (58.3%), C (75%), c (75%), *E* (25%), e (100%), KELL (5%) in the case group, and M (86.2%), N (52.3%), S (64.6%), Jka (72.3%), Jkb (67.7%), P1(72.3%), Fya (70.8%), Fyb (64.6%), Lea (18.5%), Leb (47.7%), C (73.8%), c (64.6%), E (26.2%), e (96.9%), KELL (10.8%) in the control group.

**Conclusion:**

This study provides valuable data on the distribution of minor blood group antigens in Iranian women, informing transfusion medicine and healthcare strategies. While no statistically significant differences were observed between the groups, these findings enhance our understanding of blood group prevalence in this population.

## Bullet points

- The prevalence of fertility disorders, high treatment costs, and low success rates highlight the need for identifying primary factors contributing to infertility.
- Multiple studies have shown a connection between blood groups and specific diseases.
- This study provides the first prevalence data on minor blood group antigens in infertile women in Iran.

**Abbreviations**
ART: assisted reproductive technologies
EDTA: ethylenediaminetetraacetic acid
HDFN: hemolytic disease of the fetus and newborn
IVF: in vitro fertilization
WHO: World Health Organization

## 1 Background

Infertility, which is known as the inability to conceive after 12 months or more of unprotected sexual intercourse during the fertile phase of the menstrual cycle, is considered a disease by the World Health Organization (WHO) [1, 2]. According to the WHO classification, infertility is considered one of the most significant global challenges, affecting a large population. In 2023, approximately 17.5% of the world's population—nearly one in six people—were affected by infertility [3]. Unfortunately, the infertility rate in Iran is significantly high, with approximately 20.8% reported, surpassing even the global trend of infertility [4, 5]. According to reports and research conducted on Iranian women facing fertility problems, it has been shown that these individuals suffer from social issues and may experience domestic violence, economic deprivation, social isolation, loss of social status, and marital rejection [6]. It is also believed that infertility may play a role in some health problems, as studies have shown that both men and women with infertility are at an increased risk of developing cardiovascular diseases, cancer, and mortality [7]. On the other hand, due to the high importance of reproduction and childbearing for the continuation of nations and the regulation of populations of countries, as well as the value of children economically, culturally, and socially, especially in developing countries like Iran, the issue of infertility treatment is very important. Attention and various treatments, such as in vitro fertilization (IVF), fertility improvement surgery, hormone therapy, and ovulation stimulation have been introduced so far, among which IVF is more commonly used and is the predominant treatment due to its less invasive nature and higher success rate [8]. Despite these advancements and the use of assisted reproductive technologies (ART) on a global scale, the overall burden of infertility has not significantly changed [9]. The diagnosis and treatment of infertility can be costly, and there is no single method that definitively leads to pregnancy and the birth of a healthy baby [10]. The success rate for treating infertility remains relatively constant, with approximately 25%–35% live births per cycle. This implies that the “failure rate” is around 65%–75%, which can be distressing for couples who bear the heavy financial and emotional burdens associated with this method [10]. The prevalence of fertility disorders, coupled with high treatment costs and low success rates, necessitates the identification of the primary factors contributing to individual infertility and the provision of cost‐effective and quality fertility care [9]. According to research findings, 15% of infertility cases are considered unexplained, with potential links to lifestyle and environmental factors [11]. Previous studies have suggested a potential association between blood group phenotypes and fertility‐related conditions. A higher prevalence of the Le(a − b+) phenotype (55.3%) has been reported among subfertile women, suggesting that fucosylated Lewis determinants such as Le(b) on endometrial cells may interfere with implantation [12]. Similarly, a higher prevalence of blood group O has been reported among infertile men, suggesting a possible association between the ABO blood group and male infertility [13]. However, evidence regarding the potential role of non‐ABO blood group systems in female infertility remains limited. Therefore, investigating the distribution of these antigens among infertile women may provide exploratory and hypothesis‐generating insights into their possible association with reproductive health. This phenomenon has led researchers to investigate various factors that may fall within this 15%, one of which is the discussion and examination of blood groups other than ABO, such as Rh, MNS, P1PK, Kell, Duffy, Kidd, and Lewis. Some of these blood groups, whose numbers are also increasing, have clinical significance, and their role has been identified in certain diseases. For example, the antibodies produced against the Duffy blood group, which exists on the surface of red blood cells, endothelial cells, epithelial cells, brain cells, and kidney cells, can lead to blood transfusion reactions and hemolytic disease of the fetus and newborn (HDFN) [14]. The Lewis blood group, which appears to play a role in transplant rejection, should be considered in relevant immunohematological contexts [15]. The role of the Kidd blood group in delayed hemolytic transfusion reactions and the Kell blood group, which inhibits erythropoiesis during pregnancy and causes HDFN, appear to be associated with alloantibodies of this blood group in late‐onset anemia and reticulocytopenia in the fetus [16, 17]. The significance of these blood groups in various diseases, along with the absence of a thorough examination of these blood groups in the context of infertility until now, has led to the notion of investigating the prevalence of these blood groups in infertile individuals appearing rational. Investigating this matter seems essential to prevent unnecessary treatments and their high costs, as well as to help identify unknown factors involved in infertility. Moreover, understanding the prevalence of blood group antigens across populations is vital for transfusion medicine and healthcare planning, particularly in regions with high infertility prevalence like Iran.

## 2 Methods

This descriptive cross‐sectional study used a nonprobability consecutive sampling method. The study was approved by the Ethics Committee of Ahvaz Jundishapur University of Medical Sciences (ethics approval code: IR.AJUMS.REC.1402.724). Our study was performed in line with the principles of the Declaration of Helsinki, and all methods were performed in accordance with the relevant guidelines and regulations

Our study population included women with infertility who were referred to university hospitals in Ahvaz in 2024, with an infertility diagnosis confirmed by a gynecologist. After obtaining informed consent from individuals, blood samples were collected in ethylenediaminetetraacetic acid (EDTA)‐containing tubes as an anticoagulant from each patient for serological tests. Each blood sample was tested for blood group antigens within 72 h of collection using the tube agglutination method and reagents supplied by IMMUNDIAGNOSTICA GmbH, Germany. We determined blood group antigen and phenotype frequencies in 60 infertile women in the case group and 65 women with at least one child in the control group. All participants were from Ahvaz, Iran. The sample size was calculated based on the primary objective of comparing the frequency of the Lewis blood group phenotype between infertile women and controls, using data reported in a previous study [12]. Assuming a two‐sided significance level of *α* = 0.05, a statistical power of 80%, and expected proportions of 0.809 and 0.553 in the two groups, respectively, the required sample size was estimated to be 49 participants per group. Considering a potential 20% attrition rate, the final required sample size was calculated as 60 participants per group. Accordingly, 60 infertile women and 65 fertile controls were included in the present study.

The following antigens were investigated: C, E, c, and e in the Rh system; M, N, and S in the MNS system; P1 in the P1PK system; K in the Kell system; Lea and Leb in the Lewis system; Fya and Fyb in the Duffy system; and Jka and Jkb in the Kidd system. All antigens except antigens of the Rh and Kell systems were typed directly by adding antisera to 3%–5% of RBC suspensions of blood samples according to the manufacturer's instructions for the reagents. Positive and negative control cells and check cells were used for quality controls. Antigens of the Rh and Kell systems were typed by the gel‐card method using a gel‐card reader. It is a reliable and efficient technique based on the principles of hemagglutination. All the tests were performed according to standard serological methods, including the immediate‐spin, room‐temperature, and anti‐human globulin phase.

The data were analyzed using SPSS Statistics version 27. Descriptive analyses were performed to characterized frequencies of blood group antigens and phenotypes. To compare the antigen frequencies between the case and control groups, Fisher's exact test was used, particularly when expected cell counts were small. A two‐sided *p* < 0.05 was considered statistically significant. Given the exploratory nature of the study, no adjustment for multiple comparisons was applied, and the results were interpreted accordingly.

## 3 Results

We determined blood group antigens in 125 women, including 60 infertile women with an age range of 22–45 years and a mean of 34.68 years, and 65 fertile women who had at least one child with an age range of 26–48 years and a mean of 36.22 years. The characteristics and other variables of patients are summarized in Table 1.

**Table 1:** Characteristics and other variables related to the patients.

Variable	Subdivide	No.	Percent %
Education	Uneducated or below diploma	5	8.33
	Diploma	24	40
	Bachelor's degree or higher	31	51.67
Abortion history	Yes	14	23.33
	No	46	76.67
IVF history	Yes	45	75
	No	15	25

In this study, the determination of antigens was performed using the tube agglutination method. The overall distribution of these antigens in a total of 60 infertile women was found to be M (83.3%), N (55%), s (60%), Jka (75%), Jkb (66.7%), P1 (76.7%), Lea (16.7%), Leb (58.3%), Fya (66.7%), and Fyb (73.3%). Similarly, in a control group of 65 individuals, the distribution was determined to be M (86.2%.), N (52.3%), s (64.6%), Jka (72.3%), Jkb (67.7%), P1 (72.3%), Lea (18.5%), Leb (47.7%), Fya (70.8%), and Fyb (64.6%). The results of antigen expression determination in both the patient and control groups are presented in Table 2.

**Table 2:** Antigen frequencies of MNS, Kidd, *P*, Duffy, Lewis, Rh, and Kell blood group systems.

Antigen	Patients (*n* = 60) (%)	Control group (*n* = 65) (%)	*p*‐ value	95% CI (OR)
M	83.3 (50)	86.2 (56)	0.80	0.30–2.14 (0.80)
N	55 (33)	52.3 (34)	0.85	0.55–2.25 (1.11)
S	60 (36)	64.6 (42)	0.71	0.40–1.70 (0.82)
Jka	75 (45)	72.3 (47)	0.84	0.52–2.55 (1.15)
Jkb	66.7 (40)	67.7 (44)	1.00	0.45–2.02 (0.95)
P1	76.7 (46)	72.3 (47)	0.68	0.56–2.82 (1.26)
Fya	66.7 (40)	70.8 (46)	0.70	0.39–1.76 (0.83)
Fyb	73.3 (44)	64.6 (42)	0.33	0.70–3.24 (1.51)
Lea	16.7 (10)	18.5 (12)	0.81	0.35–2.23 (0.88)
Leb	58.3 (35)	47.7 (31)	0.28	0.76–3.11 (1.54)
C	75 (45)	73.8 (48)	1.00	0.48–2.38 (1.06)
c	75 (45)	64.6 (42)	0.24	0.76–3.56 (1.64)
E	25 (15)	26.2 (17)	1.00	0.42–2.10 (0.94)
e	100 (60)	96.9 (63)	0.49	—
KELL	5 (3)	10.8 (7)	0.32	0.11–1.77 (0.44)

In the infertile group, the frequencies of the K, C, c, E, and e antigens, determined using the gel‐card technique, were 5%, 75%, 75%, 25%, and 100%, respectively. The corresponding frequencies in the control group were 10.8%, 73.8%, 64.6%, 26.2%, and 96.9%, respectively (Table 2).

Moreover, the prevalence of phenotypes associated with each of these antigens is presented in Table 3.

**Table 3:** Phenotype frequencies in MNSs, P, Duffy, Lewis, and Kidd blood group systems.

Phenotype	Patients (*n* = 60)	Control group (*n* = 65)	*p*‐value
M + N−	45% (27)	47.69% (31)	0.85
M + N+	38.33% (23)	38.46% (25)	1.00
M − N+	16.66% (10)	13.85% (9)	0.80
M − N−	0	0	—
JK a + b−	31.66% (19)	30.77% (20)	1.00
JK a + b+	43.33% (26)	41.53% (27)	0.85
JK a − b+	23.33% (14)	26.15% (17)	0.83
JK a−b−	1.66% (1)	1.53% (1)	1.00
Fy a + b−	26.66% (16)	35.38% (23)	0.33
Fy a + b+	40% (24)	35.38% (23)	0.71
Fy a−b+	33.33% (20)	29.23% (19)	0.70
Fy a−b−	0	0	—
Le a + b−	15% (9)	16.92% (11)	0.81
Le a + b+	1.66% (1)	1.53% (1)	1.00
Le a−b+	56.66% (34)	46.15% (30)	0.28
Le a−b−	26.66% (16)	35.38% (23)	0.33

Fisher's exact test revealed no statistically significant differences between the case and control groups for any of the investigated blood group antigens (all *p* > 0.05).

## 4 Discussion

Until now, several studies have demonstrated the associations between blood groups and certain diseases. The association of blood group antigens with some diseases are biologically plausible, as cell surface molecules containing blood group epitopes play a significant role in controlling the function of proteins, infections, and cancer. Therefore, their impact on susceptibility or resistance to diseases seems logical [18].

The first study that definitively reported the association between blood groups and disease was conducted by Aird and colleagues in 1953. This study specifically examined the relationship between blood group A and gastric carcinoma [19].

In a study conducted by Liu and colleagues in 2012 on breast cancer patients, it was observed that a significant number of them (59.1%) had the Fy (a − b−) phenotype, while the least observed phenotype among them was Fy (a + b+) (29.8%). However, the mechanism of the Duffy blood group in breast cancer risk has not yet been determined [20].

In another study conducted by Abdollahi et al. in 2013, it was found that there is a significant association between male infertility and blood group O, with 50% of infertile men in the study having blood group O. Furthermore, according to this study, it appears that there may be a significant relationship between this blood group and miscarriage, as the prevalence of this blood group in men whose partners have a history of miscarriage is reported to be 56% [21].

The findings of study conducted by Enevold and colleagues in 2019 suggest a relationship between the Le (a − b−) phenotype of the Lewis blood group and non‐fatal stroke [22].

In a previous study conducted among subfertile women, 55.3% of the participants were reported to have the Le(a − b+) phenotype. The authors suggested that the presence of fucosylated determinants, such as Le(b), on the surface of endometrial cells may interfere with implantation. These findings suggest that Lewis blood group phenotypes may potentially be involved in reproductive processes, particularly implantation. In contrast, in the present study, no significant differences were observed in the distribution of Lewis antigens and phenotypes between infertile and fertile women. This discrepancy may be related to differences in study populations, sample size, and other characteristics of infertility [12].

In another study conducted among infertile men in a Pakistani population, blood group O was reported to be more prevalent than the other ABO blood groups, suggesting a possible association between blood group O and male infertility. Although this finding was observed in men and involved the ABO system, it provides further evidence that blood group phenotypes may potentially be associated with reproductive health. In the present study, however, no significant differences were observed in the investigated non‐ABO blood group antigens between infertile and fertile women. The difference between these findings may be explained by differences in sex, blood group systems, study populations, and the underlying causes of infertility [13].

In a study conducted on infertile women undergoing IVF, an association was observed between ABO blood group and some fertility‐related outcomes. Women with blood group O were more likely to have elevated baseline Follicle‐stimulating hormone levels, while women with blood group A were more likely to have ovulatory infertility. In addition, women with blood group B had higher odds of live birth following IVF. These findings suggest that ABO blood groups may be associated with certain aspects of female reproductive function and IVF outcomes, although the underlying mechanisms remain unclear [23].

In another recent study conducted among infertile couples in Southern China, blood group O was found to be more prevalent among both infertile men and women compared with the reference population. The study also reported an association between ABO blood group and sperm morphology in infertile men. These findings provide further evidence that blood group may be related to certain reproductive parameters [24].

Furthermore, a multicenter case‐control study conducted in a Turkish population investigated the possible relationship between ABO blood groups and varicocele. The distribution of blood groups was generally similar between the control group and patients with unilateral varicocele; however, among patients with bilateral varicocele, blood group O was less frequent, and non‐O blood groups were more frequent than in the control group. These findings suggest that blood group may have a potential relationship with certain andrological conditions, although the observed association with varicocele does not establish a causal relationship [25].

In contrast to some previous studies, the present study did not identify significant differences in the distribution of the investigated non‐ABO blood group antigens and phenotypes between infertile and fertile women. This discrepancy may be related to differences in the blood group systems investigated, study populations, sample sizes, and characteristics or etiologies of infertility. In addition, most previous studies have focused on the ABO system, whereas the present study primarily investigated non‐ABO blood group antigens. Therefore, further large‐scale, multicenter studies are needed to clarify whether specific blood group antigens are associated with infertility or particular reproductive disorders. However, the data provide critical insights into blood group distributions among Iranian women, aligning closely with frequencies in other Asian populations, such as India (M (86.25%), N (60.9%), S (52.7%), Jka (78.55%), Jkb (66.1%), Fya (70.75%), Fyb (61.65%), C (82.6%), c (52.8%), E (20.35%), e (98.7%), and KELL (5.35%)) but differing from European populations (M (28%), N (22%), S (11%), Jka (77%), Jkb (74%), Fya (17%), Fyb (34%), C (68%), c (80%), E (29%), e (98%), and KELL (0.2%)) [26]. These variations underscore the influence of ethnic and genetic backgrounds on blood group prevalence.

## 5 Conclusion

This study provides valuable data on the distribution of non‐ABO blood group antigens among infertile and fertile women in Iran. Although no significant association was observed between the investigated blood group antigens and infertility, the findings contribute to the understanding of blood group variation in this population and provide a basis for further investigation of their potential role in reproductive health. Larger multicenter studies are warranted to further evaluate these associations.

### 5.1 Limitations

This study has some limitations that should be considered when interpreting the findings. The relatively limited sample size may have reduced the ability to detect small differences, particularly for less frequent blood group antigens and phenotypes. Detailed information on some characteristics of infertility, including its specific etiology and primary or secondary status, was not available for all participants. Similarly, some potentially relevant clinical characteristics were not systematically recorded. Finally, as several blood group antigens and phenotypes were evaluated, the findings should be considered exploratory. Further multicenter studies with larger sample sizes and more detailed clinical characterization of infertility could provide additional evidence regarding these potential associations.

## Author Contributions

**Zeinab Eftekhar:** investigation, writing – original draft, writing – review and editing, formal analysis, methodology, project administration. **Mohammad Ali Jalali Far:** visualization, validation, supervision, writing – review and editing, conceptualization, project administration. **Mehdi Torabizadeh:** methodology, data curation, conceptualization. **Najmaldin Saki:** conceptualization, supervision. **Roshan Nikbakht:** methodology, data curation.

## Funding

The authors have nothing to report.

## Ethics Statement

This study was performed in line with the principles of the Declaration of Helsinki. Approval was granted by the Ethics Committee of Ahvaz Jundishapur University of Medical Sciences with Ethics code: IR.AJUMS.REC.1402.724.

## Consent

Informed consent was obtained from all individual participants included in the study.

## Conflicts of Interest

The authors declare no conflicts of interest.

## Transparency Statement

The corresponding author, Mohammad Ali Jalali Far, affirms that this manuscript is an honest, accurate, and transparent account of the study being reported; that no important aspects of the study have been omitted; and that any discrepancies from the study as planned (and, if relevant, registered) have been explained.

## Data Availability

The data that support the findings of this study are available from the corresponding author upon reasonable request.
